# Variations in Kojic Acid Production and Corn Infection Among *Aspergillus flavus* Isolates Suggest a Potential Role as a Virulence Factor

**DOI:** 10.3390/toxins16120539

**Published:** 2024-12-13

**Authors:** Rebecca R. Sweany, Matthew K. Gilbert, Carol H. Carter-Wientjes, Geromy G. Moore, Matthew D. Lebar

**Affiliations:** Food and Feed Safety Research Unit, Southern Regional Research Center, US Department of Agriculture, New Orleans, LA 70124, USA

**Keywords:** aflatoxin, plant–fungal interactions, pathogenicity, mycotoxins, secondary metabolism, biological control, ddPCR

## Abstract

Kojic acid is a secondary metabolite with strong chelating and antioxidant properties produced by *Aspergillus flavus* and *A. oryzae*. Although antioxidants and chelators are important virulence factors for plant pathogens, the ecological role of kojic acid remains unclear. We previously observed a greater gene expression of antioxidants, especially kojic acid, by non-aflatoxigenic *A. flavus* when co-cultured with aflatoxigenic *A. flavus.* Aflatoxin production was also reduced. In this study, we investigated kojic acid production in 22 *A. flavus* isolates from Louisiana and compared them to four common *A. flavus* strains in liquid medium and on corn kernels. Corn kernel infection was assessed by quantifying the maize *beta tubulin* DNA content of the kernels using drop digital PCR (ddPCR). Maize *beta tubulin* DNA content decreased with increased corn kernel infection. Greater kojic acid production by *A. flavus* isolates coincided with greater levels of corn kernel infection. All isolates produced 60 and 700 times more kojic acid than aflatoxin and cyclopiazonic acid (a known virulence factor), respectively, which varied among sclerotial size categories. *A. flavus* strains with small sclerotia, which were rarely isolated from corn, produced the least kojic acid and infected corn kernels the least, while medium and large sclerotia strains—mainly isolated from corn—produced the most kojic acid and were more infectious. Non-aflatoxigenic isolates from Louisiana produced the most kojic acid. These results suggest that kojic acid is a potential virulence factor and may increase the pathogenic success of medium and large sclerotia-producing *A. flavus*, which could ultimately lead to more effective *A. flavus* biocontrol strains. Further studies are required to determine the effects that kojic acid has on the redox environment during corn infection and how the altered redox environment decreases aflatoxin production.

## 1. Introduction

*Aspergillus flavus* is a concern for agriculture because it can infect corn, peanuts, tree nuts, hot peppers, and cotton seed and subsequently contaminate these crops with acutely toxic and carcinogenic aflatoxin [1]. In the US, crop insurance indemnities due to the losses from the aflatoxin contamination of corn are expected to increase from USD 10 million to 31 million per year in the next 10–20 years due to the impacts of climate change [2]. To reduce the risks associated with aflatoxin and crop loss, non-aflatoxigenic (Non-tox) *A. flavus* biocontrol strains have been developed and utilized worldwide [3,4]. When used in field conditions, Non-tox strain conidiospores (conidia) are coated onto a carbon source (e.g., sterile grain), which are then deployed in corn rows prior to the mid-silking stage. Upon dispersal onto the soil surface, the Non-tox conidia proliferate, resulting in an over-representation in the *A. flavus* population [5,6,7]. This results in a greater colonization of corn kernels by Non-tox than the local aflatoxigenic (Tox) population and subsequently lowers aflatoxin contamination by 80% and higher [3,4,5,6,7]. Additionally, developing technologies such as bioengineered corn expressing antimicrobial peptides [8] or host-induced gene-silencing strategies targeting pathogenic infection [9,10,11] make it critically important to understand the mechanisms driving *A. flavus* virulence.

*Aspergillus flavus* is a highly diverse species, with variations in aflatoxin and sclerotia production. There is a diversity among Non-tox isolates, which differ in the genetic basis for lacking aflatoxin production [12]. Examples include SNP differences in one or two genes in the aflatoxin biosynthesis gene cluster and a complete deletion of genes or the entire gene cluster [12]. Sclerotia are melanized balls of mycelia that house asci and ascospores after sexual fertilization [13]. Large (L) sclerotial strains produce relatively few sclerotia, which are larger than 400 µm in diameter, produce variable quantities of aflatoxin, and are typically isolated from contaminated corn, cotton, and peanuts [14,15,16,17,18,19,20,21,22,23,24]. Non-tox L-strains typically have defects in the *aflC* (*pksA*) gene, resulting in no aflatoxin production [25,26,27,28]. Small (S) sclerotial strains produce large quantities of sclerotia that are less than 400 µm in diameter and consistently produce greater quantities of aflatoxin but are less frequently isolated from contaminated crops [14,15,16,17,18,19,20,21,22,23,24]. However, a closely related *Aspergillus* species that produces small sclerotia was identified as the causal agent of a deadly outbreak of aflatoxicosis from contaminated corn in Kenya, Africa [29]. Newly reported mixed (M) sclerotial strains produce a mixture of S and L sclerotia on Wickerham medium [28]. Many of the characterized M-strain isolates, including biocontrol isolate NRRL 21882, are missing either all or half of the aflatoxin biosynthesis gene cluster [28]. Some M-strain isolates produce small quantities of aflatoxin (<75 ppb). The M (IB) isolates are in a phylogenetically distinct cluster from L (IC) and S (IA) clusters [28,30]. Many M (IB)-clade isolates are reported to produce only L sclerotia but likely produce both S and L sclerotia [28,30,31,32]. It is challenging to understand the historical significance of the M-clade and their occurrence in corn. Recently, an M-strain vegetative compatibility group (VCG) was isolated from a single ear of corn in Louisiana in 2007 [23,28], and in Mississippi, NRRL 21882-like M-strain isolates have been frequently isolated from corn [33], suggesting that the M-strain is likely part of the *A. flavus* corn population. Although corn and soil populations fluctuate from year to year [33,34,35,36], understanding how certain Tox and Non-tox strains persist will help improve the selection of biocontrol strains and identify potential genes to target for host-induced gene silencing.

Kojic acid is a copper and iron chelator and strong antioxidant [37,38] produced by *A. flavus* and the phylogenetically similar *A. oryzae*, which is used for fermented products like shoyu (soy sauce) and alcoholic beverages like sake [39]. Kojic acid has many uses including as an antibiotic, a skin-lighting ingredient in cosmetics, and an anti-bruising agent for fresh fruits and vegetables [37,40,41,42,43,44]. Most *A. oryzae* strains are also lacking the aflatoxin biosynthetic gene cluster [39], similar to biocontrol isolates KD17 and NRRL 21882 [28]. Recent transcriptomic sequencing of the biocontrol isolate KD17 revealed that genes in the kojic acid synthesis pathway were upregulated during co-culture with Tox 53 [45]. Since aflatoxin is hypothesized to be produced under oxidative stress conditions [46,47], it was proposed that aflatoxin production was decreased during this biocontrol interaction due to kojic acid decreasing oxidative stress during cultivation. Kojic acid also has the potential to serve as a virulence factor during corn infection [48] due to its antioxidant and chelating properties [37,38]. A plant’s first line of defense against invading pathogens is an oxidative burst that can cause damage to the fungal cell wall [46,47,48,49]. As such, aflatoxin production is proposed to dampen these oxidative bursts [46,47,49]. Likewise, kojic acid may also aid in infection by changing the redox chemistry. Additionally, by chelating iron, pathogens can more effectively grow in an iron-deficient environment and starve surrounding plant cells of iron [48]. Another iron chelator produced by *A. flavus*, cyclopiazonic acid (CPA), is also proposed to be a virulence factor [50] due to its increased production in isolates from corn versus soil [23] and cytotoxicity in plants and animals.

This study was conducted to investigate kojic acid production in several Non-tox and biocontrol isolates (Af36, K49, and NRRL 21882) [5,6,7] and to determine whether it was conserved among Non-tox isolates belonging to the L and M sclerotial clades [23,28]. Additionally, this study was conducted to determine whether kojic acid produced in greater quantities by VCGs (quasi-clonal strains) was associated more with corn than soil from a Louisiana *A. flavus* population (Table 1) [23], which were previously genome-sequenced and belong to L (IC), M (IB), and S (IA) clades [28]. Finally, to further investigate the virulence potential of kojic acid, droplet digital PCR (ddPCR) was used to predict the amount of maize *beta tubulin* DNA in kernels that were inoculated with the *A. flavus* isolates and to determine whether their differential ability to colonize/infect kernels corresponded to kojic acid production.

## 2. Results

### 2.1. Isolate Selection

At least one *A. flavus* isolate from each of the 16 VCGs isolated from Louisiana soil and corn samples was selected to screen for kojic acid, aflatoxin, and cyclopiazonic acid production and corn kernel infection (Table 1). VCGs 1, 4, 7, and 10 were more frequently isolated from corn than soil samples and VCGs 2, 3, 6, 8, 11, 12, 13, 14, 15, and 16 were not recovered from corn. Nine of those isolates produced large (L) sclerotia, seven produced small (S) sclerotia, and six produced a mixture (M) of S and L sclerotia. Nine isolates produced less than 13 ppb of aflatoxin B_1_. Additionally, biocontrol Non-tox isolates NRRL 21882, AF36, and K49 were included to determine whether high kojic acid production is conserved among Non-tox isolates.

### 2.2. Kojic Acid Production in Liquid Medium

**Figure 1:**
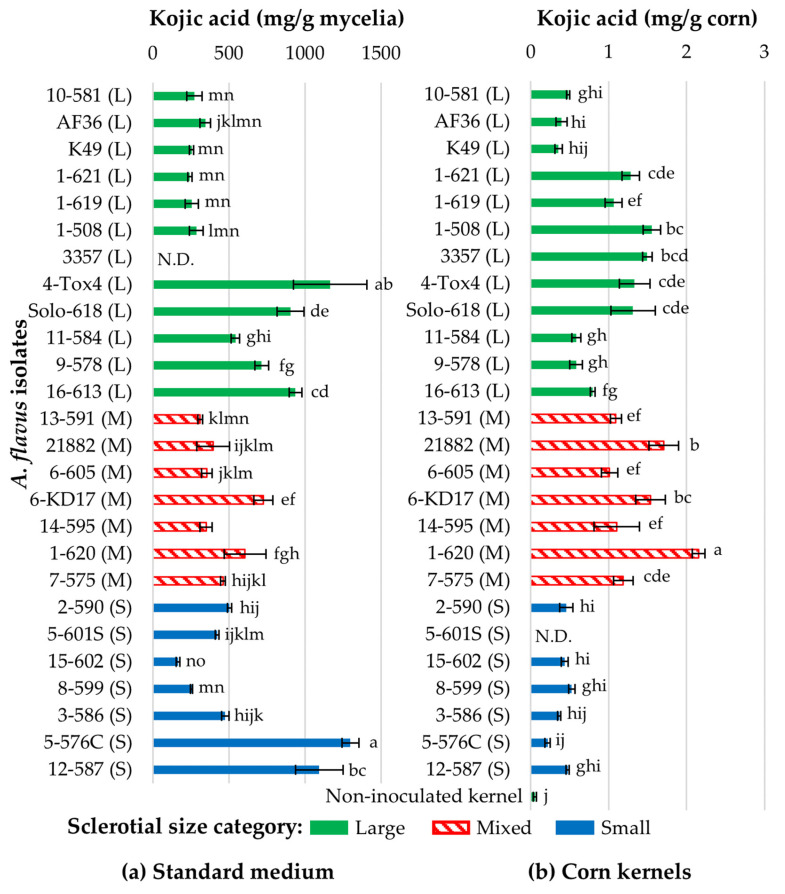
Kojic acid production by *Aspergillus flavus* isolates in (**a**) standard medium and (**b**) corn kernels. Kojic acid was measured for isolates belonging to different vegetative compatibility groups (VCGs) and with different sclerotia sizes (small (S), mixed (M), and large (L)) grown in (**a**) standard medium and (**b**) corn kernels for five and six days, respectively. The VCG determined in Sweany et al. 2011 [23] is denoted as the first number in the isolate name followed by a dash and the SRRC collection number. Isolates are ordered based on their phylogenetic similarities reported in Sweany et al. 2024 [28]. Within (**a**,**b**), means and standard error followed by the same letters are statistically similar based on preplanned comparisons of LS-means (α < 0.05) implemented in linear models. N.D. indicates that data are missing for an isolate.

To determine the levels of kojic acid production in the selected *A. flavus* isolates, liquid standard medium was inoculated with conidiospores and maintained at 30 °C for 5 days. Kojic acid was measured in the filtered culture and quantified by UPLC-MS. All *A. flavus* isolates produced high quantities of kojic acid, ranging from 250 to 1300 mg of kojic acid per g of mycelial dry weight (Figure 1a). Isolates within a sclerotial size group did not consistently produce similar amounts of kojic acid, as indicated by a significant statistical interaction (F_23,51_ = 25.96, *p* < 0.0001). Within the L-strains, 4-Tox4 which was frequently isolated from corn produced the most kojic acid of all isolates tested (1164 mg/g of fungal dry weight ± s.e. 240), whereas L-strain isolates within vegetative compatibility group (VCG) 1 which were most frequently isolated from corn and Non-tox biological control isolates K49 and AF36 produced 76% less kojic acid than 4-Tox4. Other L Tox isolates produced intermediate quantities of kojic acid. S-strain isolates 5-576C (1298 ± 57 mg/g) and 12-587 (1093 ± 156 mg/g) produced similarly high levels of kojic acid. The remaining S Tox isolates (*n* = 5) produced 70% less kojic acid than 5-576C and 12-587. The M-strain isolates, including biocontrol isolate NRRL 21882, produced kojic acid similar to the lower-producing L and S isolates with the exception of Non-tox KD17 (725.08 ± 62.56 mg/g) and Tox 1-620 (605.87 ± 137.942 mg/g), which is in VCG 1.

### 2.3. Kojic Acid Production by A. flavus During Kernel Infection

To determine the level of kojic acid produced in our *A. flavus* isolates during corn kernel infection, conidiospores in water were added to culture wells with corn kernels and incubated for 6 days. Subsequent extraction and UPLC-MS analyses indicated that isolates produced, on average, 600-fold less kojic acid in corn kernels (0.8723 ± 0.0565 mg kojic acid/g corn) than in standard medium (504.6 ± 37.8 mg of kojic acid/g fungal dry weight) (Figure 1b). L-strain isolates 1-621, 1-619, and 1-508 became more capable of producing kojic acid in corn than in standard medium, whereas S-strain isolates 5-576C and 12-578 produced less kojic acid in corn kernels than standard medium. Like in standard medium, isolates within a sclerotial size group did not consistently produce the same amount of kojic acid (F_23,51_ = 10.12, *p* < 0.0001). M-strain Tox 1-620 produced the greatest quantity of kojic acid in corn kernels (2.15 ± 0.08 mg/g), whereas M-strain isolates Non-tox KD17 (1.54 ± 0.19 mg/g) and biocontrol NRRL 21882 produced 25% less kojic acid. L-strain Tox isolate 4-Tox4 (highest producer in standard medium) and Solo-618, and Non-tox VCG1 isolates 1-508 and 1-621 (low producers in standard medium) produced similar quantities of kojic acid to KD17 and NRRL 21882. Tox L-strain isolates 11-584, 9-578, and 16-613 produced 60% less kojic acid than KD17 and NRRL 21882. Non-tox 10-581 and biocontrol isolates K49 and AF36 produced 74% less kojic acid than KD17 and NRRL 21882. S-strains, which were rarely isolated from corn, also produced 75% less kojic acid than KD17 and NRRL 21882.

### 2.4. Aflatoxin B_1_ Production

**Figure 2:**
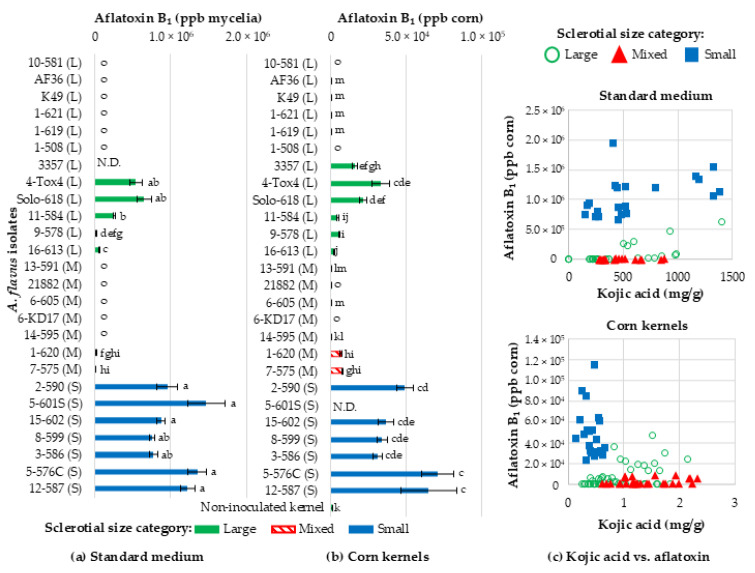
Aflatoxin B_1_ production by *Aspergillus flavus* isolates in (**a**) standard medium and (**b**) corn kernels. Aflatoxin B_1_ was measured for isolates belonging to different vegetative compatibility groups (VCGs) and with different sclerotia sizes (small (S), mixed (M), and large (L)) grown in (**a**) standard medium and (**b**) corn kernels for five and six days, respectively. The VCG determined by Sweany et al. 2011 [23] is denoted as the first number in the isolate name followed by a dash and the SRRC collection number. Isolates are ordered based on their phylogenetic similarities reported by Sweany et al. 2024 [28]. Means and standard error bars followed by the same letters are statistically similar based on preplanned comparisons of LS-means (α < 0.05) implemented in a single linear model, which included both standard medium and corn kernel aflatoxin values. N.D. indicates that data are missing for an isolate. (**c**) Kojic acid production values of each isolate are plotted against aflatoxin production values in both substrates, which did not have a linear relationship in either standard medium (F_1,33.8_ = 1.55, *p* = 0.221) or corn kernels (F_1,41.3_ = 0.00, *p* = 0.960).

Much like kojic acid production, there was a significant interaction between medium (corn or liquid standard medium), sclerotial size, isolates (F_22,132_ = 49.71, *p* < 0.0001), and aflatoxin B_1_ production (Figure 2). However, isolates generally produced more aflatoxin in standard medium than in corn kernels. S-strain isolates (1,056,192 ± 71,711 ppb and 47,789 ± 4702 ppb in standard medium and corn, respectively) and L-strain isolates 4-Tox4 and Solo (608,823 ± 62,571 ppb and 27,259 ± 3720 ppb in standard medium and corn, respectively) produced the most aflatoxin in both substrates. L-strain isolates 11-584, 9-578, and 16-613 and M-strain isolates 1-620 and 7-575 produced lower quantities of aflatoxin, especially in corn kernels. Biocontrol isolates K49, AF36, NRRL 21882, and KD17; L-strain isolates from VCGs 1 and 10; and M isolates 13-591, 6-605, and 14-595 produced no aflatoxin in standard medium. However, a few kernels were occasionally contaminated with small quantities of aflatoxin (15.7 ± 4.6 ppb).

#### Lack of Relationship Between Kojic Acid and Aflatoxin Production

On average, isolates produced 1500 and 60 times less aflatoxin (341.6 ± 56.6 ppm and 14.2 ± 21.5 ppm on standard medium and corn, respectively) than kojic acid (504,575 ± 37,799 ppm and 872.3 ± 56.5 ppm in standard medium and corn, respectively). It is noteworthy that all isolates produced at least 200 ppm of kojic acid in corn kernels and 11 isolates did not produce measurable aflatoxin. For Tox isolates, there were no significant linear relationships between kojic acid production and aflatoxin production in standard medium (F_1,33.8_ = 1.55, *p* = 0.221) or corn kernels (F_1,41.3_ = 0.00, *p* = 0.960) (Figure 2b), suggesting that fluctuations in kojic acid production did not affect aflatoxin production and vice versa. This was typified by high aflatoxin production among S-strain isolates and two L-strain isolates (4-Tox4 and Solo-618) but 70–75% lower kojic acid production by S isolates than 4-Tox4. Additionally, some Non-tox isolates produced large quantities of kojic acid, whereas AF36, K49, and 10-581 produced 74% less kojic acid than 6-KD17 and NRRL 21882.

### 2.5. Cyclopiazonic Acid Production by A. flavus During Corn Kernel Infection

**Figure 3:**
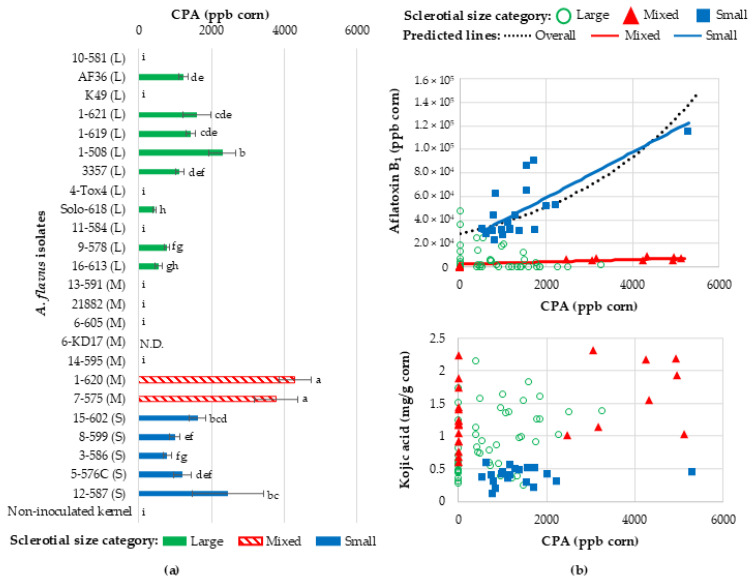
Cyclopiazonic acid production (CPA) by *Aspergillus flavus* in corn kernels. (**a**) CPA was measured for isolates belonging to different vegetative compatibility groups (VCGs) and with different sclerotia sizes (small (S), mixed (M), and large (L)) grown in corn kernels for six days. The VCG determined by Sweany et al. 2011 [23] is denoted as the first number in the isolate name followed by a dash and the SRRC collection number. Isolates are ordered based on their phylogenetic similarities reported by Sweany et al. 2024 [28]. Means and standard error followed by the same letters are statistically similar based on comparisons of LS-means (α < 0.05) implemented in linear models. N.D. indicates that data are missing for an isolate. (**b**) In the top panel, CPA values are plotted against aflatoxin for each isolate. Estimated linear relationships between CPA and aflatoxin are depicted by lines. In the bottom panel, CPA is plotted against kojic acid for individual isolates, and since there is no significant linear relationship between CPA and kojic acid, no lines are depicted.

Isolates produced on average 700-fold less cyclopiazonic acid (CPA) (1.227 ± 0.167 ppm) than kojic acid (872.3 ± 58.7 ppm) on corn kernels. Like observed with aflatoxin and kojic acid, isolates did not consistently produce the same quantities of CPA within sclerotial size groups (F_22,78_ = 439.88, *p* < 0.0001) (Figure 3). M-strains 1-620 (4297.5 ± 442.262 ppb) and 7-575 (3771.75 ± 590.812 ppb) produced the most CPA, whereas M-strains 13-591, NRRL 21882, 6-605, and 14-595 produced no measurable CPA. All S-strains produced CPA, but 35% less CPA than 1-620 and 7-575. L-strains (1178 ± 121 ppb) produced similar quantities to S-strains (1411 ± 229 ppb), except L-strains 10-581, K49, 4-Tox4, and 11-584 that did not produce any CPA.

CPA production related more with aflatoxin production than kojic acid production, which resulted in a positive linear relationship between CPA and aflatoxin (est. 0.000302 log AFB_1_ ppb/CPA ppb, F_1,82.1_ = 5.59, *p* = 0.0205) but not between CPA and kojic acid production (est. 0.1677 log ppb CPA/log mg/g kojic acid, F_1,71.6_ = 0.11, *p* = 0.7436). S-strains consistently produced both CPA and aflatoxin, and generally produced more aflatoxin than L- and M-strains; however, S-strains produced similar CPA to L CPA-producing isolates and less than M CPA-producing isolates. In contrast, S-strain isolates produced less kojic acid than M-strains and many L-strains, except L-strain isolates 10-581, K49, AF36, 11-584, Solo-618, and 9-578 that produced similar quantities of kojic acid on corn kernels to S-strains. While all 10 Non-tox isolates produced kojic acid, 7 of the 10 did not produce CPA. However, Non-tox 1-508 (2298 ppm ± 376) was the third highest producer of CPA.

### 2.6. Conidiospore Production on Corn Kernels

**Figure 4:**
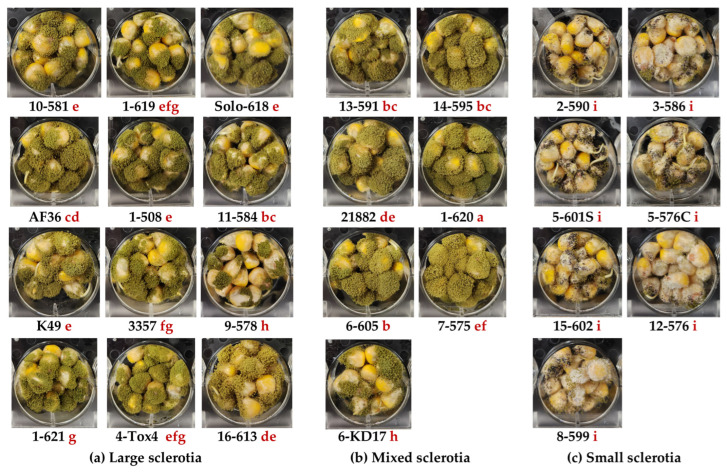
Conidiospore production of *Aspergillus flavus* isolates on corn kernels. Green conidia and black sclerotia on corn inoculated with isolates belonging to different vegetative compatibility groups (VCGs) and with different sclerotia sizes (small (S), mixed (M), and large (L)). The VCG determined by Sweany et al. 2011 [23] is denoted as the first number in the isolate name followed by a dash and the SRRC collection number. The same letter behind the isolate name refers to the mean conidial production that is statistically similar based on preplanned comparisons of LS-means (α < 0.05) implemented in linear models. Isolates are ordered in columns from top to bottom followed by rows from left to right based on their phylogenetic similarities reported by Sweany et al. 2024 [28].

There was a significant variation in spore production on corn kernels among isolates belonging to a sclerotia size class (F_2,72.5_ = 4.61, *p* = 0.0130) (Figure 4). M-strain isolates 1-620, 13-591, and 14-595 and L-strain isolate 11-584 produced the most conidiospores. Except for 6-KD17 and 7-575, M-strains produced slightly more spores (2.90 × 10^7^ ± 1.54 × 10^6^ conidia/mL) than L-strains (2.10 × 10^7^ ± 7.86 × 10^5^ conidia/mL). S-strains produced 95% and 93% fewer conidia (1.48 × 10^6^ ± 2.65 × 10^5^ conidia/mL) than M- and L-strain isolates, respectively.

#### Limited Relationship Between Kojic Acid and Spore Production

There was an overall positive linear relationship between kojic acid production and spore production (est. 9.66 × 10^6^ ± 5.30 × 10^5^ conidia/mg/g kojic acid, F_1,73.4_ = 73.4, *p* < 0.0001), which was likely driven by the low conidial and kojic acid production of S-strains compared to M- and L-strains. However, within S-strains (est. 5.32 × 10^5^ ± 1.11 × 10^6^ conidia/mg/g kojic acid, F_1,22_ = 0.23, *p* = 0.6378) and M-strains (est. 2.18 × 10^6^ ± 1.71 × 10^6^ conidia/mg/g kojic acid, F_1,22_ = 1.27, *p* = 0.2169), there were no significant linear relationships between kojic acid production and conidial production in corn kernels. Conversely, there was a significant negative linear relationship between kojic acid and conidial production for L-strains (est. −2.20 × 10^6^ ± 7.70 × 10^5^ conidia/mg/g kojic acid, F_1,35.2_ = 8.16, *p* = 0.0071), suggesting that L-strain isolates that produce higher concentrations of kojic acid produce fewer conidia.

### 2.7. Quantification of Corn Genomic DNA to Determine the Rate of Kernel Infection by A. flavus

**Figure 5:**
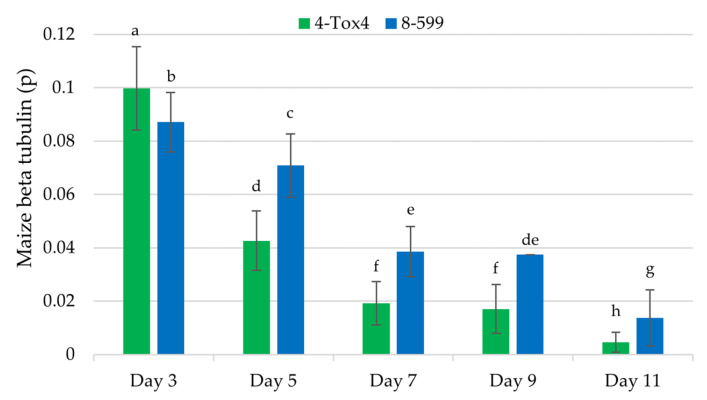
Relative levels of genomic DNA in corn kernels inoculated with *A. flavus* over an 11-day time course. Droplet digital PCR was used to amplify the target genomic sequence (*beta tubulin*), and the ratio (i.e., proportion (p)) of positive droplets that were amplified to total droplets formed was determined. The same letters reported above average ratios (p) with standard error bars are statistically similar based on comparisons of LS-means (α < 0.05) implemented in generalized linear models.

To quantify the relative levels of infection by *A. flavus* in corn kernels (i.e., rate of infection), droplet digital PCR (ddPCR) was used to detect and amplify a genomic DNA region containing the maize *beta tubulin* gene. A preliminary time course study (Figure 1) demonstrated that the ratio (i.e., proportion (p)) of positive droplets amplifying genomic DNA in the region of the maize *beta tubulin* gene to total droplets decreased with increasing time of infection (est. −0.369 ± 0.005 $\hat{p}$ beta tubulin, F_1,13_ = 5393, *p* < 0.0001 and est. −0.219 ± 0.004 $\hat{p}$ beta tubulin, F_1,11_ = 3292, *p* < 0.0001) (Figure 5). This indicates that as the fungus infects and colonizes the kernel tissue, maize DNA comprises a smaller proportion of DNA due to both growth of the fungus and rotting of the corn kernel tissue. Additionally, there was a smaller ratio of positive PCR droplets when kernels were infected with L isolate 4-Tox4 than S isolate 8-599 at each time point, indicating that 4-Tox4 better colonized and rotted corn kernel tissue. On day 9, two outliers were removed from the analysis.

**Figure 6:**
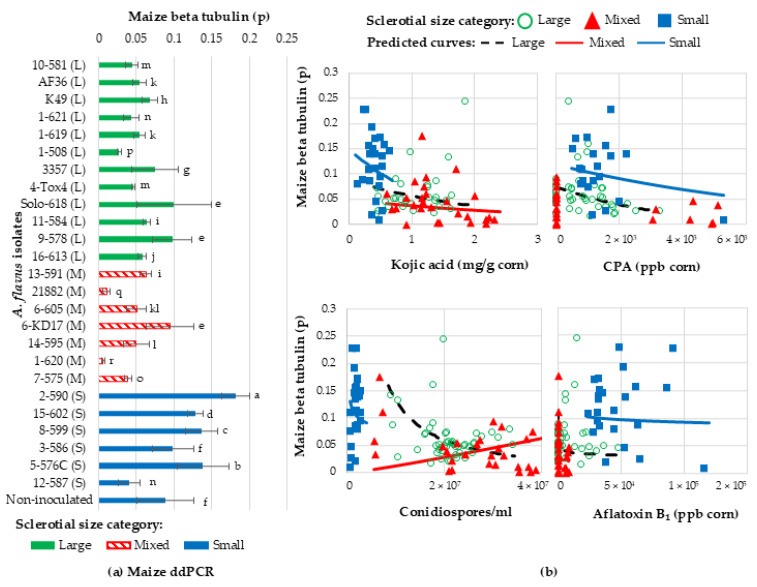
Ratio of maize gene content (p) is reduced during infection with isolates that produced more kojic acid, CPA, aflatoxin, and conidia. (**a**) The ratio of maize beta tubulin gene droplets to total droplets (p) was measured with droplet digital PCR for total DNA extracted from kernels inoculated with large (L), mixed (M), and small (S) sclerotia isolates. Smaller ratios (p) indicate less corn DNA in a sample and a greater level of corn infection. (**b**) The ratio of beta tubulin (p) was plotted against kojic acid, CPA, conidiospore, and aflatoxin production for each isolate. Estimated lines were also plotted if there was a statistically significant linear relationship between kojic acid, CPA, conidiospore, aflatoxin production, and maize beta tubulin within sclerotial size groups.

The *A. flavus* isolates examined in this study were co-incubated with corn kernels for 6 days at 30 °C. After genomic DNA extraction from the kernels, ddPCR was used to quantify corn genomic DNA content, which indicated the relative level of *A. flavus* infection. There was a significant interaction between isolates and sclerotial grouping (F_23,78_ = 719.88, *p* < 0.0001), and the level of infection (ratio of maize DNA to total droplets (p)) (Figure 6). On average, S-strains had the highest ratio of maize DNA droplets (*p* = 0.125 ± 0.012), which meant that they colonized/infected the kernels less than the L- and M-strains. Both L- and M-strains had the lowest ratios of maize DNA droplets (*p* = 0.061 ± 0.006 and *p* = 0.045 ± 0.008, respectively), which demonstrated higher levels of kernel infection. Of note, corn kernels infected with biocontrol isolate NRRL 21882 had the lowest ratio of maize DNA, which demonstrated the highest levels of infection. Of note, VCGs isolated from corn (VCG 1, 4, 7, and 10) demonstrated higher levels of corn infection than the remaining VCGs isolated from soil.

#### Relating Kojic Acid, CPA, Aflatoxin, and Spore Production with Kernel Infection

There was a significant interaction (F_2,49_ = 20.40, *p* < 0.0001) between sclerotial size, spore, kojic acid, CPA production, and ratio of corn DNA droplets, i.e., the level of *A. flavus* infection (Figure 6b). For L-strains (est. −0.46 ± 0.02 $\hat{p}$ beta tubulin/mg/g kojic acid, F_1,35_ = 406.83, *p* < 0.0001), M-strains (est. −0.30 ± 0.03 $\hat{p}$ beta tubulin/mg/g kojic acid, F_1,18_ = 145.87, *p* < 0.0001), and S-strains (est. −0.88 ± 0.06 $\hat{p}$ beta tubulin/mg/g kojic acid, F_1,17_ = 222.04, *p* < 0.0001), there was a negative relationship between kojic acid production and the ratio of corn DNA (p), which indicated that greater kojic acid production also resulted in greater *A. flavus* infection levels. There was also a negative relationship between CPA production and the ratio of corn DNA (p) for L-strain (est. −0.00034 ± 0.000021 $\hat{p}$ beta tubulin/CPA ppb, F_1,35_ = 266.91, *p* < 0.0001) and S-strain (est. −0.00015 ± 0.000011 $\hat{p}$ beta tubulin/CPA ppb, F_1,14_ = 178.84, *p* < 0.0001) isolates, which indicated that greater CPA production also resulted in greater *A. flavus* infection for L- and S-strains. However, there was no significant relationship between CPA and the ratio of corn DNA or *A. flavus* infection for M isolates (est. 0.0000051 ± 0.000028 $\hat{p}$ beta tubulin/CPA ppb, F_1,15_ = 0.03, *p* = 0.8613). Interestingly, M-strain isolates (including isolates 21882 and 6-605) had some of the highest rates of infection despite not producing any CPA. There was also a negative relationship between aflatoxin production and the ratio of corn DNA (p) L (est. −0.1016 ± 0.008190 $\hat{p}$ beta tubulin/log aflatoxin ppb, F_1,35_ = 153.77, *p* < 0.0001), M (est. −0.06414 ± 0.01000 $\hat{p}$ beta tubulin/log aflatoxin ppb, F_1,18_ = 41.11, *p* < 0.0001), and S (est. −0.07395 ± 0.02288 $\hat{p}$ beta tubulin/log aflatoxin ppb, F_1,14_ = 10.45, *p* = 0.0060) sclerotial isolates.

Much like kojic acid, CPA, and aflatoxin, there was a negative relationship between spore production and the ratio of corn DNA (p) for L-strains (est. −1.30 ± 0.05 $\hat{p}$ beta tubulin/log spore/mL, F_1,35_ = 608.11, *p* < 0.0001) and S-strains (est. −0.20 ± 0.02 $\hat{p}$ beta tubulin/log spore/mL, F_1,16_ = 136.27, *p* < 0.0001), which indicated that greater spore production also resulted in greater *A. flavus* kernel infection. In contrast, there was a positive relationship for M isolates (est. 1.19 ± 0.05, F_1,18_ = 493.03, *p* < 0.0001). The data suggest more kernel invasion and rot for L- and S-strains is associated with more visible signs of disease in the form of conidia, whereas for M-strain isolates, more visible signs of disease do not always relate to more disease or kernel invasion.

## 3. Discussion

### 3.1. Kojic Acid Production Is Associated with Greater Corn Colonization

Kojic acid is a secondary metabolite [52] produced by *Aspergillus flavus* and *A. oryzae* and other *Aspergillus* and *Penicillium* spp. [39]. It has antioxidant, antifungal, and chelating properties [37,38,39,40,41,42,43,44], but it is not understood why the fungi produce kojic acid. Since chelators and antioxidants serve as virulence factors for other plant pathogenic fungi [48,50] and may regulate aflatoxin production [46,47,49], this study examined kojic acid production among *A. flavus* biocontrol isolates and VCGs from corn and soil [23]. Here, all *A. flavus* isolates produced 60 and 700 times more kojic acid than the mycotoxin aflatoxin and phytotoxin CPA, respectively. In this study, L and M-strain sclerotial isolates from Louisiana [23,28], which were more frequently isolated from corn than S-strains, typically produced more kojic acid than S-strains. Additionally, kojic acid production had a negative relationship with intact corn kernel DNA as measured by ddPCR, suggesting that isolates with a greater ability to produce kojic acid are likely better colonizers of corn and kojic acid is a potential virulence factor. L-strain isolates from VCG 1 and 4, which were most frequently isolated from corn in Sweany et al. 2011 [23], were among the isolates that produced the most kojic acid and the least corn DNA droplets, suggesting they were readily isolated from corn because they are well adapted to infect corn. Some M-strains (NRRL 21882, 1-620, and 7-575) were more or equally capable of infecting corn, suggesting that some VCGs less readily isolated from corn in 2007 are equally specialized to infect corn and explain how different strains can predominate populations in different years [33,34,35,36]. Here, S-strain isolates produced the least kojic acid when infecting corn, which coincided with more corn DNA droplets, suggesting that the S-strains did not infect corn as well as L- and M-strains. Previously, S-strains were less invasive than cotton bolls, which was assessed using bright green-yellow fluorescence (BGYF) and is an indirect measure of kojic acid [18]. The reduced infective ability observed for S-strains, compared to the other sclerotial groups, offers a possible explanation of why S-type strains are rarely isolated from corn [14,15,16,17,18,19,20,23,31]. Despite the less invasive infection of cotton locules and corn kernels, S-strain isolates produced the most aflatoxin in both cottonseed [18] and corn. Overall, there was no linear relationship between kojic acid and the aflatoxin contamination of corn, which was consistent with the *A. flavus* infection of cotton locules [18]. Fluorescence is a powerful tool to screen kernels and other crops for aflatoxin contamination [53,54], but mostly detects kojic acid fluorescence, not aflatoxin. Kojic acid production in Aspergillus spp. that produce little to no aflatoxin is a challenge to the technology [53,54], resulting in occasional false positives, which should become more prevalent as high kojic acid-producing biocontrol strains like NRRL 21882 are used as an effective biocontrol strategy [3,4,5,6,7].

### 3.2. Kojic Acid Production Among Biocontrol Strains

In a previous study, biosynthesis genes for kojic acid and genes for other secondary metabolites with antioxidant properties were expressed at higher levels during the growth of biocontrol Non-tox isolate KD17 compared to Tox 53, which is in the same VCG as 4-Tox4 [45]. The secretion of those chemicals was suggested to reduce aflatoxin production during the biocontrol interaction by lowering oxidative stress on the Tox isolate, which minimized ROS-induced aflatoxin production [46,47,49]. Here, all Non-tox isolates tested produced kojic acid in corn kernels, suggesting that they have the capacity to alter the redox state of corn kernels and other crops. Although kojic acid production was highest in M-strain Non-tox isolates NRRL 21882 and KD17 and L-strain Non-tox members of VCG 1, closely related L-strain isolates AF36, K49, and 10-581 produced much less kojic acid. Despite lower kojic acid production, secreted chemicals (extrolites) from AF36 and K49 were found to inhibit aflatoxin similar to KD17 [55] and AF36 extrolites inhibited aflatoxin more than KD17 and NRRL 21882 on a different medium [56], suggesting that additional extrolites inhibit fungal growth or aflatoxin production. A lower production of kojic acid by L-strain isolates AF36, K49, and 10-581 (similar to S-strain isolates) is probably a relic of previous sexual reproduction between an S- and L-strain that resulted in those isolates exhibiting norB/cypA deletions consistent with S-strains, while retaining the L morphotype [25]. Previously, AF36 and NRRL 21882 did not produce kojic acid on Wickerham medium [57]. Here, greater kojic acid production may be attributable to the differences in growth on corn kernel host tissue vs. artificial medium [57], which does not always capture the full potential secondary metabolite production in crops [58,59]. Additionally, kojic acid production is affected by the extraction conditions. Kojic acid is highly hydrophilic and much more soluble in water [39] than methanol, ethyl acetate, and dichloromethane [57]. A previously reported gene expression of kojic acid synthesis genes at 30 and 72 h suggested that KD17 produced more kojic acid than Tox 54 (same VCG as 4-Tox4) [45], and after 5 or 6 days of growth in this study, 4-Tox4 produced equal or more amounts of kojic acid as KD17. However, the UPLC-MS quantification of kojic acid after 40 h of growth showed that KD17 (88.6 ± 14.5 µg/mL of broth media) produced 88× more kojic acid than 4-Tox4 (10.8 ± 2.1 µg/mL) (unpublished data). The timing of kojic acid production is likely an important factor for regulating aflatoxin production during the biocontrol interaction between Non-tox and Tox isolates. Previously, contact within the first 24 h of growth between Non-tox with Tox was required for the reduction in aflatoxin production [60]. This coincides with a 24–36 h time period where a significant upregulation of genes in the aflatoxin biosynthetic gene cluster occurs [61] and suggests that the production of inhibitory extrolites should occur early in development to fully regulate aflatoxin synthesis. It still needs to be determined if kojic acid can regulate aflatoxin production. Several recent studies found that chemicals able to reduce aflatoxin production concomitantly stimulate kojic acid production [62]. Conversely, knocking out kojic acid production stimulates the production of other secondary metabolites [63], indicating a possible compensatory interaction between kojic acid and other secondary metabolites.

### 3.3. Kojic Acid Chemical Properties May Contribute to Virulence on Corn Kernels

This study demonstrated the linear relationship between kojic acid production and kernel colonization, which provided indirect evidence that kojic acid is a virulence factor for *A. flavus*. It will be important to investigate the interactions between kojic acid and the plant oxidative defense response and other defense enzymes to establish direct evidence of kojic acid’s role in virulence. Kojic acid has the potential to be a virulence factor for the infection of corn and other crops due to its known iron- and copper-chelating ability as well as its antioxidant properties [37,38,39,40,41,42,43,44], which are properties that assist in virulence for other plant pathogens [48]. Additionally, kojic acid can act as a virulence factor by interfering with part of plants’ wound healing response through the inhibition of melanin production [37,41,42,43]. Kojic acid inhibits melanin production in plants by inhibiting copper-containing polyphenol oxidases (i.e., tyrosinase) [37,41,42,43]. Polyphenol oxidases are inhibited by limiting O_2_ uptake and reducing quinone to diphenols, which ultimately stops melanin production [37,41,42,43]. Polyphenol oxidases are also involved in plant defense against plant pathogens and insects [64,65], suggesting kojic acid has the potential to limit plant defense responses beyond melanin production. Therefore, the inhibition of polyphenol oxidases by kojic acid could allow for the greater infection of corn by interfering with both melanin and other plant defense responses, which is supported by the greater colonization of corn by *A. flavus* that produced more kojic acid. Kojic acid was produced in greater quantities than CPA, which is a known *A. flavus* virulence factor that chelates iron and is phytotoxic in corn seedling radicles [50]. Previously, VCGs isolated from corn (VCGs 1, 4, 5, 7, 9, and 10) [23] produced at least 10× greater CPA than those VCGs only isolated from soil (VCGs 2, 3, 6, 8, 11, and 14) [50]; however, isolates from VCG 10 and 4 did not produce any CPA in corn. A lack of production of CPA in corn by some VCGs commonly isolated from corn suggests that CPA is a virulence factor but is not necessary for pathogenicity on corn. Consistent with earlier reports, all S-strain isolates produced CPA and L-strain isolates varied in their abilities to produce CPA [66]. Here, M-strain isolates that retained the aflatoxin biosynthetic gene cluster also produced large quantities of CPA, while those missing either a portion or the whole cluster did not produce any CPA. Since both CPA and kojic acid are chelators, it remains unclear how these interact with other plant enzymes that require iron or copper to determine if they are detrimental to plant growth and advantageous to *A. flavus* during host–pathogen interactions.

### 3.4. M-Strains Gain Inoculum Potential on Corn Kernels

Previously, M-strains were defined by their ability to produce both S (<400 µm) and L (>400 µm) sclerotia and produce those at intermediate densities [28]. Additionally, M-strains produced intermediate quantities of conidia [28]. In this study, M-strain isolates produced as many, if not more, conidia than L-strains on corn kernels, and as expected, they produced a greater abundance of conidia than the S-strains. This clarifies conflicting studies between *A. flavus* populations in Louisiana [23,28], Mississippi, and elsewhere [33] where many NRRL 21882-like M-strains were found in Mississippi and the US but rarely in Louisiana corn. This suggests that both M and L-strains have a similarly high inoculum potential on corn and explains why both are readily isolated from corn. To more fully understand *A. flavus* population dynamics, especially with respect to the isolates’ ability to infect crops, it is important to investigate the potential for conidia production on crop debris and plants in surrounding fields. NRRL 21882 and other M-strains (e.g., 7-575 and 1-620) were the best infectors of corn, which suggests the M-strains are well adapted to inhabit the corn niche. Since the M-strains produce either no aflatoxin, or much less than widespread L-strain 4-Tox4, more studies are needed to understand the pathological success of 4-Tox4.

## 4. Conclusions

This study presents evidence that kojic acid production is likely a virulence factor that leads to greater corn infection by *Aspergillus flavus*. *A. flavus* L- and M-strains produce more kojic acid and were more frequently isolated from corn, while S-strains, which are less frequently isolated from crops [14,15,16,17,18,19,20,23,31], produced less kojic acid and infected corn to a lesser extent. While kojic acid is a strong antioxidant [37,38], its potential roles in counteracting the plants’ oxidative burst during defense [46,47,49] and aflatoxin production [46,47] are not known. All Non-tox isolates produced kojic acid but it is still unclear whether this antioxidant production contributes to the ability of Non-tox biocontrol strains to colonize crops and regulate aflatoxin production during kernel infection. It is also unclear whether kojic acid produced by *A. flavus* during infection is involved in inhibiting plant phenol oxidases [37,41,42,43], which plants use for defense [64,65]. The results reported here are promising, but further research is needed to determine whether higher kojic acid production can improve the effectiveness of new biocontrol *A. flavus* against aflatoxin-producing strains.

## 5. Materials and Methods

### 5.1. Fungal Strains/Isolates

Most of the *A. flavus* isolates used in this study belong to 16 different vegetative compatibility groups (VCGs) from Louisiana [23], which are dispersed among sclerotial size groups (L, M, and S) [28]. These isolates, along with several Non-tox (biocontrol) isolates, were evaluated for the production of kojic acid, cyclopiazonic acid (CPA), aflatoxin, and conidia on corn kernels and kojic acid and aflatoxin in liquid standard medium [44]. Isolate information is shown in Table 1. All isolates are housed in a publicly available culture collection located at the Southern Regional Research Center (USDA-ARS) in New Orleans, Louisiana. For each experiment, conidia were harvested in ultra-purified deionized water from one-week-old cultures grown in darkness at 30 °C on 2× V8-medium (100 mL of V8 (Campbell Soup Co., Camden, NJ, USA), 900 mL of H_2_O, and 20 g of bacteriological-grade agar (VWR International, Randor, PA, USA); pH adjusted to 5.2).

### 5.2. Growth Conditions for Liquid Medium Experiments

Isolates belonging to 16 different VCGs and sclerotial size groups (L (*n* = 12), M (*n* = 7), and S (*n* = 7)) were grown in liquid standard medium with slight modifications from Terabayashi et al. 2010 [44] in a completely randomized design with three biological reps per isolate. In 6-well plates (353046 Falcon, Corning Inc., Corning, NY, USA), 5 mL of standard medium (0.25% yeast extract, 0.1% K_2_HPO_4_, 0.05% MgSO_4_–7H_2_O, and 10% glucose; pH adjusted to 6.0) was added to each well. Each isolate was inoculated with 5 × 10^5^ conidia in 3 wells for 3 independent biological replicates in a single 6-well plate to avoid potential strain-to-strain volatile interactions which can reduce growth and alter secondary metabolism [67,68]. The inoculated 6-well plates were incubated in separate boxes atop two wetted paper towels and randomly arranged in a dark 30 °C incubator for five days. Mycelia were harvested from each well and dried on chromatography paper in a biosafety cabinet. Aflatoxin and kojic acid were analyzed from the remaining medium below the mycelial mats in each well.

### 5.3. Growth Conditions for Corn Kernel Experiments

Isolates belonging to different VCGs and sclerotial size groups (L (*n* = 12), M (*n* = 7), and S (*n* = 7)) were grown on corn kernels in a similar manner to Mehl and Cotty 2013 [58] with slight modifications in a completely randomized design with four biological reps per isolate. In 6-well plates, 5 g of untreated, non-GMO, yellow, dent-corn kernels of H97C (Hybrid85, Omaha, NE, USA) were inoculated with 1 mL of 0.5 × 10^5^ conidia/mL to give an approximate 25% moisture content in each well. Each isolate was inoculated on corn kernels in four wells for four independent biological replicates in a single 6-well plate to avoid strain-to-strain volatile interactions [67,68]. The inoculated 6-well plates were incubated in separate boxes atop two wetted paper towels and randomly arranged in a dark 30 °C incubator for six days. Conidia were harvested from corn kernels by shaking them in 20 mL of water twice, and then counted using a model R1 digital cell counter (Olympus Corp., Tokyo, Japan). Kernels were frozen on dry ice and stored at −80 °C. Kernels were ground with a Geno/Grinder 2010 (SPEX SamplePrep, Metuchen, NJ, USA) for 4 min at 1700 rpm. Kojic acid, CPA, aflatoxin, and DNA were extracted from corn kernel powder as described below.

### 5.4. Kojic Acid Analysis

Standard medium samples (50 µL of medium) from each of the three replicate well cultures per isolate were diluted 20-fold in water and centrifuged to pelletize particulates [40,42]. Ground corn samples (200 mg) from each of the four replicate wells containing corn per isolate were extracted with 1 mL of water (LC/MS grade) on a shaker (200 rpm) overnight. The extracts were centrifuged to pelletize the particulates and the supernatant was diluted 20-fold with LC/MS-grade water [40]. Kojic acid (KA) analysis was conducted on a Waters Acquity UPLC system (Waters Corp, Milford, MA, USA) coupled to a Waters Xevo G2 XS QTOF mass spectrometer (Waters Corp, Milford, MA, USA) for each sample. Injections (1 µL) were separated on a Waters BEH C18 1.7 µm, 2.1 × 50 mm column (Waters Corp, Milford, MA, USA) with the following solvent system: 0.5 mL/min, solvent A: 0.1% formic acid (Fisher Scientific Co., Hampton, NH, USA) in water; solvent B: 0.1% formic acid in acetonitrile (Fisher Scientific Co., Hampton, NH, USA); pump settings: isocratic at 100% A for 2.5 min (0–2.5 min), gradient to 95% A over 0.5 min (2.5–3.0 min), gradient to 80% A over 1.0 min (3.0–4.0 min), isocratic at 80% A for 1 min (4.0–5.0 min), and then column equilibration to 100% A for 2.5 min (5.0–7.5 min). The Z-spray ionization source was run in ESI+ mode using MassLynx 4.2 software (Waters Corp, Milford, MA, USA) with the following spectrometer settings: source temperature: 100 °C, desolvation temperature: 250 °C, desolvation gas flow: 600 L/h, cone gas flow: 50 L/h, capillary voltage: 3.0 kV, and sampling cone voltage: 40 V. Analyses were performed in sensitivity and continuum mode, with a mass range of m/z 50–1200 and a scan time of 0.1 s. A data-independent acquisition method with elevated collision energy (MSE) was used with a low energy of 6 eV and a high energy ramp from 15 to 45 eV. Mass data were collected for the first 2 min of the 7.5 min run, imported into Waters UNIFI 1.9.4 software (Waters Corp, Milford, MA, USA), and quantified using the “Quantify Assay Tof 2D” analysis method with the lock mass corrected by UNIFI. Kojic acid was purchased from Sigma-Aldrich (St. Louis, MO, USA) and used for quantification. Kojic acid content is expressed in ppm (µg kojic acid/g seed).

### 5.5. Aflatoxin and Cyclopiazonic Acid Analysis

Liquid standard medium samples (1 mL) from each of the three replicate well cultures per isolate were mixed with ethyl acetate and the upper solvent layer was concentrated and resuspended in 1 mL of methanol (Fisher Scientific Co., Hampton, NH, USA). The ground corn samples (200 mg) from each of the four replicate wells containing corn per isolate were extracted with 1 mL of methanol on a shaker (200 rpm) overnight. The redissolved extracts from both substrates were centrifuged to pelletize the particulates and the supernatant was analyzed as is or diluted 10-fold with methanol (Fisher Scientific Co., Hampton, NH, USA), if necessary, to avoid detector saturation. Aflatoxin analysis was conducted on a Waters Acquity UPLC system (Waters Corp, Milford, MA, USA) coupled to a Waters Acquity fluorescence detector (Ex = 365 nm, Em = 440 nm) (Waters Corp, Milford, MA, USA) [67] for each sample. Injections (1 µL) were chromatographed with a 40% methanol (Fisher Scientific Co., Hampton, NH, USA)/60% water isocratic solvent system on the same column described above used for KA analysis. Aflatoxin B_1_ (AFB_1_) and Aflatoxin B_2_ (AFB_2_) standards (Sigma-Aldrich, St. Louis, MO, USA) were used for identification and quantification. Aflatoxin content is expressed in ppb (ng AF/g seed). Cyclopiazonic acid (CPA) analysis was conducted on the methanol extracts using the same Waters system, mass spectrometer settings, and column that were used for KA analysis above, but a different solvent gradient: (1 µL injections, 0.5 mL/min, solvent A: 0.1% formic acid (Fisher Scientific Co., Hampton, NH, USA) in water; solvent B: 0.1% formic acid in acetonitrile (Fisher Scientific Co., Hampton, NH, USA)): 5% B isocratic for 1.25 min (0–1.25 min), gradient to 25% B over 0.25 min (1.25–1.5 min), gradient to 100% B over 4.5 min (1.5–5.0 min), 100% B isocratic for 2.5 min (5.0–7.5 min), and then column equilibration to 5% B for 2.5 min (7.5–10 min) [67]. Data were analyzed and quantified using Waters UNIFI 1.9.4 software (Waters Corp, Milford, MA, USA) as described for KA analysis. CPA was purchased from Sigma-Aldrich (St. Louis, MO, USA) and used for quantification. CPA content is expressed in ppb (ng CPA/g seed).

### 5.6. DNA Analysis Using Droplet Digital PCR

DNA was extracted from 50 mg of yellow, dent-corn kernels of H97C (Hybrid85, Omaha, NE, USA) samples using a Wizard Purification kit (Promega, Madison, WI, USA) according to their protocol with slight modifications reported below [23,69]. Ground corn was homogenized with microbeads in 600 µL of nuclei lysis buffer with a Geno/Grinder for 2 min in a pre-chilled (−20 °C) block using Synergy homogenization tubes (OPS Diagnostics, Lebanon, NJ, USA). A second protein precipitation step was included by adding 100 µL of protein precipitation solution and incubating for 5 min.

To establish the proof of concept that droplet digital PCR (ddPCR) technology can be utilized to quantify kernel colonization by *A. flavus*, approximately 5 g of kernels of H97C (Hybrid85, Omaha, NE, USA) was added to 1 well of a 6-well tissue culture plate and inoculated with 1 × 10^6^ conidia in 1 mL of water from *A. flavus* isolates 4-Tox4 and 8-599. The plates were stored on top of damp paper towels in sealed Tupperware containers. The containers were stored at 30 °C in 12 h light/dark cycles. After 3, 5, 7, 9, or 11 days of incubation, kernels were removed and shaken in 20 mL of H_2_O multiple times to remove exterior fungal spores and wiped with a Kim wipe before being stored at −80 °C. Kernels were then pulverized while frozen using a Geno/Grinder. DNA was extracted using the protocol described above. All samples were repeated in triplicate. To quantify kernel colonization for other isolates in this study, DNA was obtained from corn kernels inoculated in Section 5.3.

ddPCR was conducted according to the manufacturer’s instructions (BioRad, La Jolla, CA, USA). Briefly, QX200 ddPCR EvaGreen Supermix (Cat # 186-4034, BioRad, La Jolla, CA, USA) was combined with 100 nM primers and 60 ng of DNA. To quantify maize DNA, maize-specific beta tubulin primers (5′-TACCTCACCTGCTCTGCTAT-3′, 5′-GACGAAGTAGGTCTGGTTCTTG-3′) were utilized. After droplet formation, cycle parameters were as follows: 95 °C for 5 min, cycle 40 times at 95 °C for 30 s and 57 °C for 1 min, and then 4 °C and 90 °C for 5 min and finally 4 °C for infinity. The Droplet Reader was operated with default settings. Data analysis was conducted using the QX Manager v2.1.0 Software. Automatic thresholds were established by Settings Auto with Tilt for the majority of samples. In the few cases that could not be determined, a manual threshold was set in the largest space between zero emittance and full emittance. Data are expressed as the percentage of total droplets determined to be positive, with the standard error calculated from at least 3 replicates.

### 5.7. Data Analysis

Linear models (i.e., analysis of variance) were used to estimate the fixed effects of the *A. flavus* isolate, sclerotial size, and their interaction on each response variable: kojic acid, aflatoxin, CPA, and conidia (Proc mixed, SASv9.4, SAS Institute, Cary, NC, USA). Aflatoxin and CPA values were log-adjusted to improve normality as tested by the Shapiro–Wilk test for normality. Preplanned comparisons between isolates (due to a significant interaction between the isolate and sclerotia) were calculated using the maximum likelihood estimation of least squares (LS) means (α < 0.05) [70].

Linear mixed models (i.e., regression analysis) were used to estimate the effects of kojic acid on aflatoxin, CPA, and conidia with sclerotial size as a fixed effect and isolate treated as a random effect. An additional linear model tested the effect of CPA on aflatoxin with sclerotial size as a fixed effect and isolate treated as a random effect. These models were also estimated for each sclerotial size group. CPA and aflatoxin values were log-transformed to improve normality as tested by the Shapiro–Wilk test for normality.

Generalized linear models (i.e., multiple contingency tables) were used to estimate the fixed effects of the *A. flavus* isolate, sclerotial size, and their interaction on the response variable ratio (p) of positive PCR droplets for maize beta tubulin to total droplets [71]. Within GLIMMIX, the logit link (log odds) and binomial distribution were utilized. The log odds was estimated as follows:

odds = p positive maize beta tubulin PCR droplet/p negative maize beta tubulin PCR droplet

p (proportion) = number positive or negative PCR droplets/total droplets

Generalized linear mixed models (i.e., logistic regression) were used to estimate the effects of kojic acid, CPA, log aflatoxin, and log conidia on p-positive PCR droplets for maize beta tubulin with sclerotial size as the fixed effect and the isolate as the random effect. The logit link and binomial distribution were utilized.

## Figures and Tables

**Table 1:** *Aspergillus flavus* isolates used in this study.

Study Names ^1^	Isolate ^2^	SRRC ^3^	Parish ^4^	Source ^5^	VCG ^6^	Sclerotia ^7^	MAT ^8^	AFB1 ^9^
1-508	07-C-7-10-3	580	St. Landry	Corn	1 (483:4)	Large	*M1-2*	0
1-620	07-M-S-3-3-2	620	St. Landry	Soil	1 (483:4)	Mixed	*M1-1*	3
1-619	07-C-1-1-4	619	Franklin	Corn	1 (483:4)	Large	*M1-2*	40
1-621	07-C-7-6-4	621	St. Landry	Corn	1 (483:4)	Large	*M1-2*	2
2-590	07-S-2-5-7	590	Franklin	Soil	2 (0:29)	Small	*M1-2*	14,793
3-586	07-S-2-1-1	586	Franklin	Soil	3 (0:6)	Small	*M1-1*	24,241
4-Tox4	07-C-1-1-1 (Tox4/LA2)	573	Franklin	Corn	4 (56:5)	Large	*M1-2*	43,145
5-576C	07-C-3-5-5	576	Tensas	Corn	5 (1:10)	Small	*M1-2*	ND
5-601S	07-S-4-4-1	601	Tensas	Soil	5 (1:10)	Small	*M1-1*	26,846
6-KD17	07-S-3-1-6 (KD17/LA1)	1588	Tensas	Soil	6 (0:5)	Mixed	*M1-2*	0
6-605	07-S-6-4-1	605	Point Coupee	Soil	6 (0:5)	Mixed	*M1-2*	4
7-575	07-C-3-2-5	575	Tensas	Corn	7 (10:2)	Mixed	*M1-1*	6
8-599	07-S-3-2-4 (LA3)	599	Tensas	Soil	8 (0:14)	Small	*M1-1*	14,653
9-578	07-C-5-1-5	578	Concordia	Corn	9 (2:14)	Large	*M1-1*	1884
10-581	07-M-C-3-1-7	581	Point Coupee	Corn	10 (10:2)	Large	*M1-2*	0
11-584	07-S-1-1-7	584	Franklin	Soil	11 (0:2)	Large	*M1-2*	11,174
12-587	07-S-2-1-2	587	Franklin	Soil	12 (0:4)	Small	*M1-2*	50,050
13-591	07-S-3-1-1	591	Tensas	Soil	13 (0:3)	Mixed	*M1-1*	13
14-595	07-S-3-1-8	595	Tensas	Soil	14 (0:2)	Mixed	*M1-2*	2
15-602	07-S-5-1-6	602	Concordia	Soil	15 (0:3)	Small	*M1-2*	29,236
16-613	07-M-S-2-4-2	613	Rapides	Soil	16 (0:2)	Large	*M1-2*	10,317
Solo-618	07-M-S-3-6-1	618	Point Coupee	Soil	Solo (0:1)	Large	*M1-1*	24,236
3357	NRRL 3357	167	Georgia	Peanut		Large	*M1-1*	ND
21882	NRRL 21882	1534	Georgia	Peanut		Mixed	*M1-2*	0
AF36	AF36	1533	Arizona	Cotton	Af36	Large	*M1-2*	0
K49	K49	1587	Mississippi	Corn	Af36	Large	*M1-2*	0

^1^ Names used throughout this study include VCG (where assessed) determined in Sweany et al. 2011 [23] as the first number followed by a dash and the SRRC number or commonly used names in the scientific literature. All data presented here are reported in Sweany et al. 2024 [28]. ^2^ Original names and (alternative names) for the majority of isolates collected in 2007 [23]. ^3^ Accession number for isolates deposited in the Southern Regional Research Center collection. ^4^ Parishes (counties) in Louisiana or state where samples were collected. ^5^ Isolation source: corn kernels, peanuts, cottonseed, or soil. ^6^ VCGs determined in Sweany et al. 2011 [23]. The numbers refer to that study only. Values in parentheses indicate the number of isolates from corn/soil. ^7^ Sclerotium diameters are noted as large (L) if all of a strain’s sclerotia are >400 μm and small (S) if all are <400 μm. Strains noted as mixed produced equivalent numbers of small and large sclerotia as reported in [28]. ^8^ Mating-type idiomorphs determined by PCR amplification as reported in [51]. ^9^ Concentration of aflatoxin B_1_ (in ppb) produced while the isolate was grown on rice; ND indicates that data are missing for an isolate.

## Data Availability

The original contributions presented in the study are included in the article, further inquiries can be directed to the corresponding authors.

## References

[1] Richard J.L. (2008). Discovery of aflatoxins and significant historical features. Toxin Rev..

[2] Yu J., Hennessy D.A., Tack J., Wu F. (2022). Climate change will increase aflatoxin presence in US corn. Environ. Res. Lett..

[3] Bandyopadhyay R., Ortega-Beltran A., Akande A., Mutegi C., Atehnkeng J., Kaptoge L., Senghor A.L., Adhikari B.N., Cotty P.J. (2016). Biological control of aflatoxins in Africa: Current status and potential challenges in the face of climate change. World Mycotoxin J..

[4] Moral J., Garcia-Lopez M.T., Camiletti B.X., Jaime R., Michailides T.J., Bandyopadhyay R., Ortega-Beltran A. (2020). Present status and perspective on the future use of Aflatoxin Biocontrol products. Agronomy.

[5] Cotty P.J., Antilla L., Wakelyn P.J., Vincent C., Goettel M.S., Lazarovits G. (2007). Competitive exclusion of aflatoxin producers: Farmer-driven research and development. Biological Control: A Global Perspective.

[6] Dorner J.W., Cole R.J., Connick W.J., Daigle D.J., McGuire M.R., Shasha B.S. (2003). Evaluation of biological control formulations to reduce aflatoxin contamination in peanuts. Biol. Control.

[7] Weaver M.A., Abbas H.K. (2019). Field displacement of aflatoxigenic *Aspergillus flavus* strains through repeated biological control applications. Front. Microbiol..

[8] Rajasekaran K., Sayler R.J., Sickler C.M., Majumdar R., Jaynes J.M., Cary J.W. (2018). Control of *Aspergillus flavus* growth and aflatoxin production in transgenic maize kernels expressing a tachyplesin-derived synthetic peptide, AGM182. Plant Sci..

[9] Gilbert M.K., Majumdar R., Rajasekaran K., Chen Z.-Y., Wei Q., Sickler C.M., Lebar M.D., Cary J.W., Frame B.R., Wang K. (2018). RNA interference-based silencing of the alpha-amylase (amy1) gene in *Aspergillus flavus* decreases fungal growth and aflatoxin production in maize kernels. Planta.

[10] Raruang Y., Omolehin O., Hu D., Wei Q., Han Z.Q., Rajasekaran K., Cary J.W., Wang K., Chen Z.-Y. (2020). Host induced gene silencing targeting *Aspergillus flavus* aflM reduced aflatoxin contamination in transgenic maize under field conditions. Front. Microbiol..

[11] Raruang Y., Omolehin O., Hu D., Wei Q., Promyou S., Parakattil L.J., Rajasekaran K., Cary J.W., Wang K., Chen Z.-Y. (2023). Targeting the *Aspergillus flavus p2c* gene through host-induced gene silencing reduces *A. flavus* infection and aflatoxin contamination in transgenic maize. Front. Plant Sci..

[12] Adhikari B.N., Bandyopadhyay R., Cotty P.J. (2016). Degeneration of aflatoxin gene clusters in *Aspergillus flavus* from Africa and North America. AMB Express.

[13] Horn B.W., Moore G.G., Carbone I. (2009). Sexual reproduction in *Aspergillus flavus*. Mycologia.

[14] Abbas H.K., Weaver M.A., Zablotowicz R.M., Horn B.W., Shier W.T. (2005). Relationships between aflatoxin production and sclerotia formation among isolates of *Aspergillus* section *Flavi* from the Mississippi Delta. Eur. J. Plant Pathol..

[15] Agbetiameh D., Ortega-Beltran A., Awuah R.T., Atehnkeng J., Islam M.-S., Callicott K.A., Cotty P.J., Bandyopadhyay R. (2019). Potential of atoxigenic *Aspergillus flavus* vegetative compatibility groups associated with maize and groundnut in Ghana as biocontrol agents for aflatoxin management. Front. Microbiol..

[16] Atehnkeng J., Ojiambo P.S., Donner M., Ikotun T., Sikora R.A., Cotty P.J., Bandyopadhyay R. (2008). Distribution and toxigenicity of *Aspergillus* species isolated from Maize kernels in three agro-ecological zones in Nigeria. Int. J. Food Microbiol..

[17] Camiletti B.X., Moral J., Asensio C.M., Torrico A.K., Lucini E.I., Giménez-Pecci M.P., Michailides T.J. (2018). Characterization of Argentinian endemic *Aspergillus flavus* isolates and their potential use as biocontrol agents for mycotoxins in maize. Phytopathology.

[18] Cotty P.J. (1989). Virulence and cultural characteristics of two *Aspergillus flavus* strains pathogenic on cotton. Phytopathology.

[19] Giorni P., Magan N., Pietri A., Bertuzzi T., Battilani P. (2007). Studies on *Aspergillus* section *Flavi* isolated from maize in northern Italy. Int. J. Food Microbiol..

[20] Mauro A., Battilani P., Callicott K.A., Giorni P., Pietri A., Cotty P.J. (2013). Structure of an *Aspergillus flavus* population from maize kernels in northern Italy. Int. J. Food Microbiol..

[21] Novas M.V., Cabral D. (2002). Association of mycotoxin and sclerotia production with compatibility groups in *Aspergillus flavus* from peanut in Argentina. Plant Dis..

[22] Pildain M.B., Vaamonde G., Cabral D. (2004). Analysis of population structure of *Aspergillus flavus* from peanut based on vegetative compatibility, geographic origin, mycotoxin and sclerotia production. Int. J. Food Microbiol..

[23] Sweany R.R., Damann K.E., Kaller M.D. (2011). Comparison of soil and corn kernel *Aspergillus flavus* populations: Evidence for niche specialization. Phytopathology.

[24] Vaamonde G., Patriarca A., Fernández Pinto V., Comerio R., Degrossi C. (2003). Variability of aflatoxin and cyclopiazonic acid production by *Aspergillus* section *flavi* from different substrates in Argentina. Int. J. Food Microbiol..

[25] Chang P.-K., Abbas H.K., Weaver M.A., Ehrlich K.C., Scharfenstein L.L., Cotty P.J. (2012). Identification of genetic defects in the atoxigenic biocontrol strain *Aspergillus flavus* K49 reveals the presence of a competitive recombinant group in field populations. Int. J. Food Microbiol..

[26] Chang P.-K., Horn B.W., Dorner J.W. (2005). Sequence breakpoints in the Aflatoxin Biosynthesis gene cluster and flanking regions in nonaflatoxigenic *Aspergillus flavus* isolates. Fungal Genet. Biol..

[27] Ehrlich K.C., Cotty P.J. (2004). An isolate of *Aspergillus flavus* used to reduce aflatoxin contamination in cottonseed has a defective polyketide synthase gene. Appl. Microbiol. Biotechnol..

[28] Sweany R.R., Mack B.M., Gebru S.T., Mammel M.K., Cary J.W., Moore G.G. (2024). Divergent *Aspergillus flavus* corn population is composed of prolific conidium producers: Implications for saprophytic disease cycle. Mycologia.

[29] Probst C., Callicott K.A., Cotty P.J. (2012). Deadly strains of Kenyan *Aspergillus* are distinct from other aflatoxin producers. Eur. J. Plant Pathol..

[30] Geiser D.M., Pitt J.I., Taylor J.W. (1998). Cryptic speciation and recombination in the aflatoxin-producing fungus *Aspergillus flavus*. Proc. Natl. Acad. Sci. USA.

[31] Drott M.T., Fessler L.M., Milgroom M.G. (2019). Population subdivision and the frequency of aflatoxigenic isolates in *Aspergillus flavus* in the United States. Phytopathology.

[32] Drott M.T., Satterlee T.R., Skerker J.M., Pfannenstiel B.T., Glass N.L., Keller N.P., Milgroom M.G. (2020). The frequency of sex: Population genomics reveals differences in recombination and population structure of the aflatoxin-producing fungus *Aspergillus flavus*. mBio.

[33] Weaver M.A., Callicott K.A., Mehl H.L., Opoku J., Park L.C., Fields K.S., Mandel J.R. (2022). Characterization of the *Aspergillus flavus* population from highly aflatoxin-contaminated corn in the United States. Toxins.

[34] Bayman P., Cotty P.J. (1991). Vegetative compatibility and genetic diversity in the *Aspergillus flavus* population of a single field. Can. J. Botechnol..

[35] Molo M.S., White J.B., Cornish V., Gell R.M., Baars O., Singh R., Carbone M.A., Isakeit T., Wise K.A., Woloshuk C.P. (2022). Asymmetrical lineage introgression and recombination in populations of *Aspergillus flavus*: Implications for biological control. PLoS ONE.

[36] Ortega-Beltran A., Cotty P.J. (2018). Frequent shifts in *Aspergillus flavus* populations associated with maize production in Sonora, Mexico. Phytopathology.

[37] Chen J.S., Wei C.-I., Marshall M.R. (1991). Inhibition mechanism of kojic acid on polyphenol oxidase. J. Agric. Food Chem..

[38] Murakami Y. (1962). Complexing behaviour of kojic acid with metal ions—II: Fe(III) chelates. J. Inorg. Nucl. Chem..

[39] Watarai N., Yamamoto N., Sawada K., Yamada T. (2019). Evolution of *Aspergillus oryzae* before and after domestication inferred by large-scale comparative genomic analysis. DNA Res..

[40] Chang P.-K., Scharfenstein L.L., Mahoney N., Kong Q. (2023). Kojic acid gene clusters and the transcriptional activation mechanism of *Aspergillus flavus* KojR on expression of clustered genes. J. Fungi.

[41] He M., Zhang J., Li N., Chen L., He Y., Peng Z., Wang G. (2024). Synthesis, anti-browning effect and mechanism research of kojic acid-coumarin derivatives as anti-tyrosinase inhibitors. Food Chem..

[42] Moon K.M., Kwon E.B., Lee B., Kim C.Y. (2020). Recent trends in controlling the enzymatic browning of fruit and vegetable products. Molecules.

[43] Son S.M., Moon K.D., Lee C.Y. (2001). Inhibitory effects of various anti browning agents on apple slices. Food Chem..

[44] Terabayashi Y., Sano M., Yamane N., Marui J., Tamano K., Higa Y., Ohashi S., Koike H., Machida M. (2010). Identification and characterization of genes responsible for biosynthesis of kojic acid, an industrially important compound from *Aspergillus oryzae*. Fungal Genet. Biol..

[45] Sweany R.R., Mack B.M., Moore G.G., Gilbert M.K., Cary J.W., Lebar M.D., Rajasekaran K., Damann K.E. (2021). Genetic responses and aflatoxin inhibition during co-culture of aflatoxigenic and non-aflatoxigenic *Aspergillus flavus*. Toxins.

[46] Baidya S., Duran R.M., Lohmar J.M., Harris-Coward P.Y., Cary J.W., Hong S.-Y., Roze L.V., Linz J.E., Calvo A.M. (2014). *VeA* is associated with the response to oxidative stress in the aflatoxin producer *Aspergillus flavus*. Eukaryot. Cell.

[47] Fountain J.C., Scully B.T., Chen Z.-Y., Gold S.E., Glenn A.E., Abbas H.K., Lee R.D., Kemerait R.C., Guo B. (2015). Effects of hydrogen peroxide on different toxigenic and atoxigenic isolates of *Aspergillus flavus*. Toxins.

[48] Zhang N., NurAinIzzati M.Z., Scher K., Condon B.J., Horwitz B.A., Turgeon B.G. (2013). Iron, oxidative stress, and virulence: Roles of iron-sensitive transcription factor Sre1 and the redox sensor ChAp1 in the maize pathogen *Cochliobolus heterostrophus*. Mol. Plant-Microbe Interact..

[49] Lanubile A., Maschietto V., Battilani P., Marocco A. (2017). Infection with toxigenic and atoxigenic strains of *Aspergillus flavus* induces different transcriptional signatures in maize kernels. J. Plant Interact..

[50] Chalivendra S.C., DeRobertis C., Chang P.-K., Damann K.E. (2017). Cyclopiazonic acid is a pathogenicity factor for *Aspergillus flavus* and a promising target for screening germplasm for ear rot resistance. Mol. Plant-Microbe Interact..

[51] Ramirez-Prado J.H., Moore G.G., Horn B.W., Carbone I. (2008). Characterization and population analysis of the mating-type genes in *Aspergillus flavus* and *Aspergillus parasiticus*. Fungal Genet. Biol..

[52] Uka V., Cary J.W., Lebar M.D., Puel O., De Saeger S., Diana Di Mavungu J. (2020). Chemical repertoire and biosynthetic machinery of the *Aspergillus flavus* secondary metabolome: A review. Compr. Rev. Food Sci. Food Saf..

[53] Hruska Z., Yao H., Kincaid R., Brown R.L., Bhatnagar D., Cleveland T.E. (2017). Temporal effects on internal fluorescence emissions associated with aflatoxin contamination from corn kernel cross-sections inoculated with toxigenic and atoxigenic *Aspergillus flavus*. Front. Microbiol..

[54] Kaklan H., Güneş A., Durmuş E., Kuşcu A. (2014). Non-invasive detection of aflatoxin-contaminated figs using fluorescence and multispectral imaging. Food Addit. Contam. Part A Chem. Anal. Control Expo. Risk Assess..

[55] Sweany R.R., DeRobertis C.D., Kaller M.D., Damman K.E. (2022). Intraspecific growth and aflatoxin inhibition responses to atoxigenic *Aspergillus flavus*: Evidence of secreted, inhibitory substances in biocontrol. Phytopathology.

[56] Moore G.G., Lebar M.D., Sweany R.R., Lohmar J.M., Carter-Wientjes C.H. (2024). Production of inhibitory extrolites is a shared trait among non-aflatoxigenic *Aspergillus flavus*. J. Appl. Microbiol..

[57] Uka V., Moore G.G., Arroyo-Manzanares N., Nebija D., De Saeger S., Diana Di Mavungu J. (2019). Secondary metabolite dereplication and phylogenetic analysis identify various emerging mycotoxins and reveal the high intra-species diversity in *Aspergillus flavus*. Front. Microbiol..

[58] Mehl H.L., Cotty P.J. (2013). Influence of plant host species on intraspecific competition during infection by *Aspergillus flavus*. Plant Pathol..

[59] Probst C., Cotty P.J. (2012). Relationships between *in vivo* and *in vitro* aflatoxin production: Reliable prediction of fungal ability to contaminate maize with aflatoxins. Fungal Biol..

[60] Huang C., Jha A., Sweany R., DeRobertis C., Damann K.E. (2011). Intraspecific aflatoxin inhibition in *Aspergillus flavus* is thigmoregulated, independent of vegetative compatibility group and is strain dependent. PLoS ONE.

[61] Chanda A., Roze L.V., Linz J.E. (2010). A possible role for exocytosis in aflatoxin export in *A. parasiticus*. Eukaryote Cell.

[62] Zhang J.D., Han L., Yan S., Liu C.M. (2014). The non-metabolizable glucose analog D-glucal inhibits Aflatoxin Biosynthesis and promotes kojic acid production in *Aspergillus flavus*. BMC Microbiol..

[63] Dao T.T., de Mattos-Shipley K.M.J., Prosser I.M., Williams K., Zacharova M.K., Lazarus C.M., Willis C.L., Bailey A.M. (2021). Cleaning the cellular Factory–Deletion of McrA in *Aspergillus oryzae* NSAR1 and the generation of a novel kojic acid deficient strain for cleaner heterologous production of secondary metabolites. Front. Fungal Biol..

[64] Taranto F., Pasqualone A., Mangini G., Tripodi P., Miazzi M.M., Pavan S., Montemurro C. (2017). Polyphenol oxidases in crops; biochemical, physiological and genetic aspects. Int. J. Mol. Sci..

[65] Zhang J., Sun X. (2021). Recent advances in polyphenol oxidase-mediated plant stress responses. Phytochemistry.

[66] Chang P.-K., Horn B.W., Dorner J.W. (2009). Clustered genes involved in cyclopiazonic acid production are next to the Aflatoxin Biosynthesis gene cluster in *Aspergillus flavus*. Fungal Genet. Biol..

[67] Moore G.G., Lebar M.D., Carter-Wientjes C.H. (2022). Cumulative Effects of Non-Aflatoxigenic *Aspergillus flavus* Volatile Organic Compounds to Abate Toxin Production by Mycotoxigenic Aspergilli. Toxins.

[68] Sweany R.R., Damann K.E. (2020). Influence of neighboring clonal-colonies on aflatoxin production by *Aspergillus flavus*. Front. Microbiol..

[69] Aime M.C., Phillips-Mora W. (2005). The causal agents of witches’ broom and frosty pod rot of cacao (chocolate, *Theobroma cacao*) form a new lineage of Marasmiaceae. Mycologia.

[70] Midway S., Robertson M., Flinn S., Kaller M. (2020). Comparing multiple comparisons: Practical guidance for choosing the best multiple comparisons test. PeerJ.

[71] Matthijs Vynck M., Vandesompele J., Nijs N., Menten B., De Ganck A., Olivier Thas O. (2016). Flexible analysis of digital PCR experiments using generalized linear mixed models. Biomol. Detect. Quantif..

